# Management and outcomes of refractory immune thrombocytopenia in pediatric patients: A retrospective analysis from Ospedale Pediatrico Bambino Gesù

**DOI:** 10.1007/s00277-025-06542-4

**Published:** 2025-10-03

**Authors:** Gabriele Canciani, Anna Cascone, Antonio Musolino, Antonio Torelli, Emanuela Monteferrario, Giuseppe Palumbo, Giulia Ceglie

**Affiliations:** 1 Department of Hematology, Oncology and Cell and Gene Therapy, IRCCS Bambino Gesù Children’s Hospital, Rome, Italy; 2 Residency School of Pediatrics, University of Rome Tor Vergata, Rome, Italy; 3 Department of Systems Medicine, University of Rome Tor Vergata, Rome, Italy

**Keywords:** Pediatric immune thrombocytopenia, Refractory immune thrombocytopenia, Romiplostim, Mycophenolate mofetil, Rituximab.

## Abstract

Immune thrombocytopenia (ITP) in pediatrics is typically self-limiting, yet a subset develops refractory ITP (rITP), which persists despite standard first-line treatments. Clinical evidence on the efficacy of second-line therapies in this condition, such as mycophenolate mofetil (MMF), rituximab (RTX) and romiplostim, remains limited. This study aims to evaluate the efficacy of second-line therapies for the management of rITP in a cohort of 33 pediatric patients treated at Ospedale Pediatrico Bambino Gesù over a 16-year period. Some of these patients switched second-line therapies due to inadequate clinical responses, thus receiving more than one treatment over the study period. Collectively, patients in our cohort received MMF (*n* = 20), RTX (*n* = 16), romiplostim (*n* = 15), dapsone (*n* = 3) and sirolimus (*n* = 2). Response rates were 50% (10/20) for mycophenolate mofetil (MMF), 18.8% (3/16) for rituximab, and 66.7% (10/15) for romiplostim. None of the 3 patients treated with dapsone showed clinical benefits. One of the two patient undergoing sirolimus achieved a sustained clinical response. Combination therapies showed promising results, particularly the association of MMF and romiplostim. Our results provide new insights into the management of pediatric rITP, while highlighting the need for further studies to establish evidence-based treatment guidelines and improve outcomes of this condition.

## Introduction

Immune thrombocytopenia (ITP) is an immune-mediated disorder characterized by isolated thrombocytopenia, resulting from increased platelet destruction and impaired platelet production. The incidence ranges from 1.9 to 6.4 per 100.000 annually [1]. Pediatric ITP typically follows a benign and self-limiting course, with less than 20% of children developing chronic ITP [2, 3]. However, a subset of patients develops ITP refractory to first line therapies (rITP), characterized by persistent thrombocytopenia and bleeding symptoms despite standard first-line treatments.

Currently, there is no universally accepted definition of rITP in children, and management strategies vary widely due to the lack of consensus and standardized guidelines [4, 5]. The International Working Group (IWG) and the 2011 American Society of Hematology (ASH) guidelines both include the failure of splenectomy as a criterion, which is less applicable to pediatric cases [3, 5]. The new Associazione Italiana Ematologia Oncologia Pediatrica (AIEOP) guidelines provide a broader definition, considering rITP in children with persistent, clinically significant bleeding and low platelet counts despite multiple treatments [6]. This aligns better with clinical practice, where splenectomy is rare in pediatric patients.

Second-line therapies for pediatric rITP include agents such as romiplostim, rituximab (RTX), and mycophenolate mofetil (MMF), among others. These treatments are often chosen based on clinical experience rather than robust evidence, as randomized controlled trials in this population are limited [6, 7].

This study aims to contribute to the limited body of evidence on pediatric rITP by evaluating the efficacy and safety of second-line treatments in a cohort of children treated at Ospedale Pediatrico Bambino Gesù (OPBG) over a 16-year period.

## Materials and methods

This retrospective observational study included patients younger than 18 years diagnosed with rITP between 2015 and 2023 at OPBG. Refractory ITP was defined as a platelet count persistently less than 20 × 10^9/L after initial treatments with intravenous immunoglobulins, corticosteroids, and eltrombopag (intended as no response after 4 weeks of Eltrombopag at a maximum dose of 75 mg per os daily). Patients with inherited platelet disorders and secondary ITP associated with autoimmune diseases were excluded from the study. Clinical data, including sex, age, complete blood counts, and treatment regimens, were collected from medical records.

### Response Criteria

- **Complete Response (CR)**: Platelet count > 100 × 10^9/L without bleeding.
- **Partial Response (PR)**: Platelet count between 30 × 10^9/L and 100 × 10^9/L without bleeding.
- **Non-Responder (NR)**: Platelet count < 30 × 10^9/L.
- **Relapse**: Platelet count to < 20 × 10^9/L, after an initial response.

## Results and discussion

### Population

**Fig. 1:**
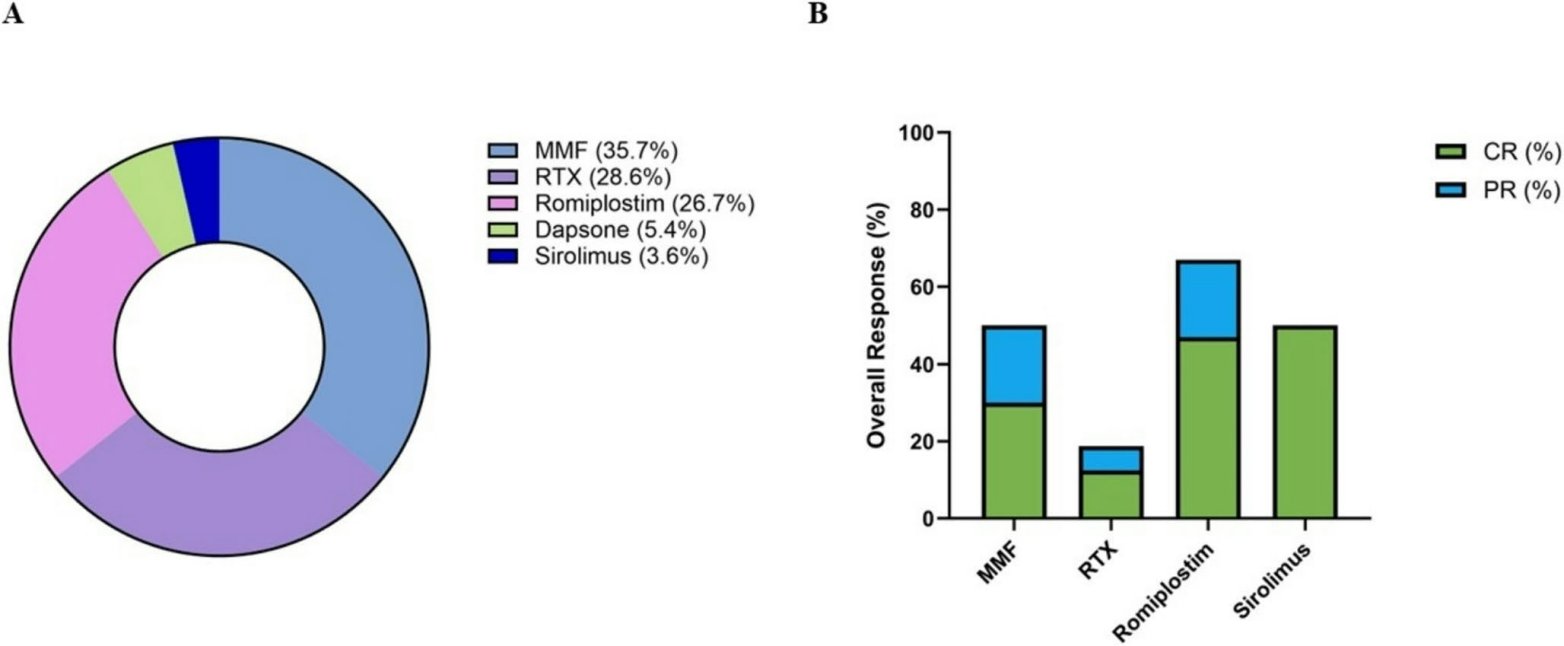
**A** Distribution of second-line treatments administered in our cohort (*n* = 56). **B** Clinical responses of patients treated with MMF, RTX, romiplostim and sirolimus; dapsone was not included in this graph as none of the treated patients exhibited a clinical response. Abbreviations: mycophenolate mofetil (MMF); rituximab (RTX); complete response (CR); partial response (PR)

We retrospectively evaluated 257 pediatric patients diagnosed with ITP in our hospital. Of these, 33 (12.8%) rITP following first-line therapies. This proportion is partially consistent with previous reported rates, although it might be influenced by referral bias associated with our tertiary care center [8]. Our study included these 33 pediatric patients with rITP, comprising 17 females (51.5%) and 16 males (48.5%). The median age at diagnosis was 5.8 years (range 1.3–16.2 years). The mean platelet count at diagnosis was 7.6 × 10^9/L (range 1–27 × 10^9/L), with a mean MPV of 9.7 fL (range 5.2–13.6 fL). Second-line treatments included MMF (*n* = 20), RTX (*n* = 16), romiplostim (*n* = 15), dapsone (*n* = 3) and sirolimus (*n* = 2) (Fig. 1A) (Table 1). None of our patients who responded to treatments showed significant bleeding. Notably, some patients underwent switching between second-line therapies due to inadequate clinical responses or treatment inefficacy observed over the study period. Thus, the cumulative total of second-line therapies administered (*n* = 56) exceeds the number of patients in the cohort.

**Table 1:** Clinical features and outcomes of pediatric rITP patients treated with second-line treatments. Abbreviations: mycophenolate mofetil (MMF); rituximab (RTX); platelets (PLTs); mean platelet volume (MPV); overall response (OR); complete response (CR); partial response (PR)

	MMF	RTX	Romiplostim	Dapsone	Sirolimus
**Number of patients**	20	16	15	3	2
**Median age at diagnosis (years)**	7 (1.3–16.2)	4.8 (1.3–12.9)	3.8 (1.3–13.4)	8.3 (3.8–12.9)	2.9 (2.3–3.5)
**Females**	10 (50%)	9 (56%)	8 (53%)	1 (33%)	1 (50%)
**Males**	10 (50%)	7 (44%)	7 (47%)	2 (66%)	1 (50%)
Mean PLTs count at diagnosis (n × 109/L)	8 (1–36)	7.5 (1–27)	7.6 (1–27)	8 (7–9)	15.5 (4–27)
**Mean MPV at diagnosis (fL)**	9.1 (5.2–13.6)	10.8 (8.3–13.6)	10.2 (8.1–13.6)	10.5 (10.1–11)	12.3 (11-13.6)
**Median time from diagnosis to initiation of** **therapy** (months)	13.7 (1.8-153.8)	15.8 (2.1–54.5)	13.5 (1.4-168.7)	66.3 (25–110)	15.6 (13.3–18)
**Co-administered therapies**	Eltrombopag (*n* = 3); Romiplostim (*n* = 2)	Eltrombopag (*n* = 1)	MMF (*n* = 2)	No	No
**OR**	10 (50%)	3 (18.75%)	10 (66.7%)	0	1 (50%)
**CR**	6 (30%)	2 (12.5%)	7 (46.7%)	0	1 (50%)
**PR**	4 (20%)	1 (6.25%)	3 (20%)	0	No
**Durable Response (> 6 months)**	5 (50% of OR)	3 (100% of OR)	8 (80% of OR)	0	1 (100% of OR)
**Toxicity**	1 (Unspecified)	Yes (Fever and Erythema)	No	No	No

### Mycophenolate mofetil

In total, 20 patients with rITP in our study cohort received MMF treatment, at a daily dosage of 900 mg/m2, divided into 2 administrations. The average time between disease onset and initiation of therapy was 34.5 months (ranging from 0.2 to 153 months). Among these patients, 50% (10/20) were responders, with two of them receiving a combination of MMF and romiplostim. Specifically, 60% (6/10) of MMF responders displayed a CR, whereas 40% (4/10) attained a PR (Fig. 1B). Moreover, 50% of responders (5/10) showed a durable response up to 6 months. The periodic therapeutic drug monitoring was performed in 17 patients. We did not observe differences in the median levels of MMF blood levels between responders (*n* = 10) and non-responders (*n* = 7) at 2 weeks (2.08 vs. 2.83 ug/mL, *p* > 0.05), 1 month (2.02 vs. 2.1 ug/mL, *p* > 0.05), 2 months (2.3 vs. 2.3 ug/mL, *p* > 0.05) ug/L), 3 months (2.81 vs. 2.7 ug/mL, *p* > 0.05) from the beginning of the treatment. One patient stopped the treatment due to unspecified side effects, while another patient died during the follow-up period due to hypovolemic shock resulting from severe menorrhagia.

### Rituximab

Sixteen patients were treated with RTX, administered as 4 weekly infusions of 375 mg/m². The treatment was started after a median time of 15.8 months (range 2.1–54.5 months) from the onset of ITP. The overall response rate was 18.8% (3/16), with 12.5% (2/16) achieving PR and 6.3% (1/16) obtaining CR (Fig. 1B). Responders maintained therapeutic benefits through a six-month follow-up. Adverse events included transient erythema (*n* = 2) and fever (*n* = 1).

### Romiplostim

Romiplostim was administered to 15 patients in our cohort, with a median time from disease onset of 13.5 months (range, 1.4–168.7 months). Among treated patients, 66.7% (10/15) exhibited a clinical response, with 70% of them achieving CR (7/10) and 30% displaying PR (3/10) (Fig. 1B). 80% of responder patients (8/10) achieved a response after 8 weeks of therapy. Only 20% of patients (2/10) experienced a disease relapse after 6 months, defined as a decline in platelet count below 20 × 10⁹/L despite ongoing treatment.

### Combination therapies

In total, 6 patients in our population received combination therapy. Two patients received MMF combined with romiplostim and 100% (2/2) achieved a CR. Among the 3 patients treated with MMF together with eltrombopag 66% (2/3) achieved a CR. The single patient who received therapy with eltrombopag and RTX did not respond.

### Others

Two patients were treated with sirolimus, at a dosage of 2 mg/m^2^, one of whom showed a durable response after failure of RTX, MMF, and romiplostim, while the other showed no response (Fig. 1B). Three patients received dapsone, but it did not yield clinical responses.

## Discussion

Our study aligns with literature demonstrating the efficacy of MMF for treating rITP. Previous studies report response rates of 52% and 64% in adult and pediatric populations, respectively [9, 10]. Higher response rates up to 81% are noted in specific populations like those with ALPS-like disorder or Evans syndrome [9, 10]. Goldberg and Levy found a 73% response rate in pediatric patients refractory to first-line therapy, with manageable adverse events and successful tapering off MMF for complete responders [11].

The ASH 2019 guidelines recommend RTX for rITP in specific cases, such as failure of first-line treatments and thrombopoietin receptor agonists, non-life-threatening mucosal bleeding, or diminished quality of life [2, 7]. Response rates to RTX in pediatric patients range from 23 to 69%, influenced by factors like patient demographics, prior therapies, and disease duration [12]. In our study, the response rate was lower at 18.8%, which could be attributed to the advanced disease state and multiple prior therapies in our cohort.

Romiplostim demonstrated a robust response rate in our study, consistent with other reports in pediatric rITP. Studies by Pasquet et al. and Bussel et al. also supported its efficacy, with response rates of 50% and comparable proportions of complete responses (70% among responders) [13, 14]. These findings underscore the efficacy and tolerability of romiplostim as an effective treatment for pediatric rITP, highlighting its potential role in managing this challenging condition. Further research is warranted to refine treatment protocols and validate long-term outcomes.

The combination of MMF and romiplostim also appeared effective, suggesting potential benefits from multimodal therapy.

Sirolimus inhibits the mTOR pathway, crucial for B- and T-lymphocyte proliferation, offering targeted immunosuppression beneficial in immune dysregulation disorders like ITP, as highlighted in recent literature [15, 16]. Moreover, sirolimus promotes regulatory T-cell proliferation, further supporting its efficacy in challenging cases of refractory ITP [17, 18].

Dapsone, considered for rITP, did not yield responses in our cohort of three patients. While valued for its affordability and safety, its effectiveness varies widely among studies and patient groups [19–21]. Its slower onset and limited efficacy in some cases underscore the importance of tailored patient selection and exploration of alternative therapies for refractory ITP.

Although this study’s retrospective nature and small sample size limit the generalizability of the findings, our analysis sheds light on managing ITP refractory to first line therapies in pediatric patients, highlighting challenges in defining and treating this condition with limited evidence-based therapies. Moving forward, prospective, large-scale studies are crucial to refine treatment strategies, validate predictors of treatment response, and enhance therapeutic outcomes while minimizing risks. This approach will improve care quality for pediatric patients with refractory immune thrombocytopenia.

## Data Availability

All data from will be available upon reasonable request to the corresponding author.

## References

[1] Terrell DR, Beebe LA, Vesely SK et al (2010) The incidence of immune thrombocytopenic purpura in children and adults: a critical review of published reports. Am J Hematol 85:174–180. 10.1002/ajh.21616 20131303 10.1002/ajh.21616

[2] Neunert C, Terrell DR, Arnold DM et al (2019) American society of hematology 2019 guidelines for immune thrombocytopenia. Blood Adv 3:3829–3866. 10.1182/bloodadvances.2019000966 31794604 10.1182/bloodadvances.2019000966 PMC6963252

[3] Neunert C, Lim W, Crowther M et al (2011) The American society of hematology 2011 evidence-based practice guideline for immune thrombocytopenia. Blood 117:4190–4207. 10.1182/blood-2010-08-302984 21325604 10.1182/blood-2010-08-302984

[4] Terrell DR, Neunert CE, Cooper N et al (2020) Immune thrombocytopenia (ITP): current limitations in patient management. Med (Kaunas) 56:667. 10.3390/medicina56120667 10.3390/medicina56120667 PMC7761470 33266286

[5] Rodeghiero F, Stasi R, Gernsheimer T et al (2009) Standardization of terminology, definitions and outcome criteria in immune thrombocytopenic purpura of adults and children: report from an international working group. Blood 113:2386–2393. 10.1182/blood-2008-07-162503 19005182 10.1182/blood-2008-07-162503

[6] Russo G, Parodi E, Farruggia P et al (2024) Recommendations for the management of acute immune thrombocytopenia in children. A Consensus Conference from the Italian Association of Pediatric Hematology and Oncology. Blood Transfus 22:253–265. 10.2450/BloodTransfus.501 10.2450/BloodTransfus.501 PMC11073630 37677093

[7] Cines DB, Blanchette VS (2002) Immune thrombocytopenic purpura. N Engl J Med 346:995–1008. 10.1056/NEJMra010501 11919310 10.1056/NEJMra010501

[8] Pincez T, Fernandes H, Fahd M et al (2024) Pediatric refractory chronic immune thrombocytopenia: identification, patients’ characteristics, and outcome. Am J Hematol 99:1269–1280. 10.1002/ajh.27337 38651646 10.1002/ajh.27337

[9] Taylor A, Neave L, Solanki S et al (2015) Mycophenolate mofetil therapy for severe immune thrombocytopenia. Br J Haematol 171:625–630. 10.1111/bjh.13622 26250874 10.1111/bjh.13622

[10] Miano M, Ramenghi U, Russo G et al (2016) Mycophenolate mofetil for the treatment of children with immune thrombocytopenia and Evans syndrome. A retrospective data review from the Italian association of paediatric haematology/oncology. Br J Haematol 175:490–495. 10.1111/bjh.14261 27447678 10.1111/bjh.14261

[11] Goldberg TA, Levy CF (2023) Mycophenolate mofetil use in pediatric immune thrombocytopenia refractory to first-line therapy: a single-center experience. J Pediatr Hematol Oncol 45:339–343. 10.1097/MPH.0000000000002688 37314887 10.1097/MPH.0000000000002688

[12] Grace RF, Bennett CM, Ritchey AK et al (2012) Response to steroids predicts response to rituximab in pediatric chronic immune thrombocytopenia. Pediatr Blood Cancer 58:221–225. 10.1002/pbc.23130 21674758 10.1002/pbc.23130 PMC3863944

[13] Pasquet M, Aladjidi N, Guiton C et al (2014) Romiplostim in children with chronic immune thrombocytopenia (ITP): the French experience. Br J Haematol 164:266–271. 10.1111/bjh.12609 24152194 10.1111/bjh.12609

[14] Bussel JB, Hsieh L, Buchanan GR et al (2015) Long-term use of the thrombopoietin-mimetic Romiplostim in children with severe chronic immune thrombocytopenia (ITP). Pediatr Blood Cancer 62(2):208–213. 10.1002/pbc.25136 25345874 10.1002/pbc.25136 PMC4309514

[15] Miano M, Rotulo GA, Palmisani E et al (2018) Sirolimus as a rescue therapy in children with immune thrombocytopenia refractory to mycophenolate mofetil. Am J Hematol 93:E175–E177. 10.1002/ajh.25119 29675829 10.1002/ajh.25119

[16] Acar SO, Tahta N, Al IO et al (2024) Sirolimus is effective and safe in childhood relapsed-refractory autoimmune cytopenias: a multicentre study. Scand J Immunol e13376. 10.1111/sji.13376 38741164 10.1111/sji.13376

[17] Battaglia M, Stabilini A, Migliavacca B et al (2006) Rapamycin promotes expansion of functional CD4 + CD25 + FOXP3 + regulatory T cells of both healthy subjects and type 1 diabetic patients. J Immunol 177:8338–8347. 10.4049/jimmunol.177.12.8338 17142730 10.4049/jimmunol.177.12.8338

[18] Stallone G, Infante B, Di Lorenzo A et al (2016) mTOR inhibitors effects on regulatory T cells and on dendritic cells. J Transl Med 14:152. 10.1186/s12967-016-0916-7 27245075 10.1186/s12967-016-0916-7 PMC4886438

[19] Rodrigo C, Gooneratne L (2013) Dapsone for primary immune thrombocytopenia in adults and children: an evidence-based review. J Thromb Haemost 11:1946–1953. 10.1111/jth.12371 23927583 10.1111/jth.12371

[20] Patel AP, Patil AS (2015) Dapsone for immune thrombocytopenic purpura in children and adults. Platelets 26:164–167. 10.3109/09537104.2014.886677 24512442 10.3109/09537104.2014.886677

[21] Damodar S, Viswabandya A, George B et al (2005) Dapsone for chronic idiopathic thrombocytopenic purpura in children and adults–a report on 90 patients. Eur J Haematol 75:328–331. 10.1111/j.1600-0609.2005.00545.x 16146539 10.1111/j.1600-0609.2005.00545.x

